# Microfluidics-Assisted Formulation of Polymeric Oxytocin Nanoparticles for Targeted Brain Delivery

**DOI:** 10.3390/pharmaceutics17040452

**Published:** 2025-04-01

**Authors:** Emmanuel Adediran, Sharon Vijayanand, Akanksha Kale, Mahek Gulani, Jennifer C. Wong, Andrew Escayg, Kevin S. Murnane, Martin J. D’Souza

**Affiliations:** 1 Nanotechnology Laboratory, Center for Drug Delivery Research, College of Pharmacy, Mercer University, Atlanta, GA 30341, USA; emmanuel.adediran@live.mercer.edu (E.A.); sharon.c.p.vijayanand@live.mercer.edu (S.V.); akanksha.madhav.kale@live.mercer.edu (A.K.); mahekanil.gulani@live.mercer.edu (M.G.); 2 Department of Human Genetics, Emory University School of Medicine, Atlanta, GA 30322, USA; jennifer.c.wong@emory.edu (J.C.W.); aescayg@emory.edu (A.E.); 3 Department of Pediatrics, Emory University and Children’s Healthcare of Atlanta, Atlanta, GA 30322, USA; 4 Department of Pharmacology, Toxicology & Neuroscience, Louisiana State University Health Sciences Center, Shreveport, LA 71103, USA; kevin.murnane@lsuhs.edu

**Keywords:** microfluidics, nanoparticles, targeted brain delivery, oxytocin, formulation

## Abstract

**Background:** The neuropeptide oxytocin has been identified as a potential therapeutic molecule. However, the therapeutic potential of this molecule is limited due to the challenges faced in oxytocin delivery to the brain. Scientific innovation has led to the breakthrough discovery of many modalities to encapsulate molecules for targeted drug delivery, which can enhance oxytocin delivery to the brain. This research aimed to explore a microfluidics-based system that optimizes the formulation of cross-linked bovine serum albumin (BSA) nanoparticles encapsulating oxytocin. **Methods**: First, the formulation parameters were optimized using a design of experiments (DOE) by evaluating the effect of flow rate, polymer concentration, and the binary solvent mixture polarity on the nanoparticle size. Drug encapsulation efficiency, release, and kinetics profile were characterized. These oxytocin nanoparticles were conjugated to rabies virus glycoprotein (RVG), a brain-targeting ligand, and the conjugation efficiency was determined. **Results**: The sizes of the nanoparticles were between 50 nm and 75 nm with a <0.4 polydispersity index. The encapsulation efficiency was >80%. Approximately 58% of oxytocin was released from the nanoparticles within the first six hours, showing an initial burst that is ideal for seizure control and thereafter exhibiting the Korsmeyer–Peppas release kinetics. **Conclusions**: For the first time, we demonstrated the microfluidics method of formulating nanoparticles with particle size of less than 100 nm, with improved encapsulation efficiency and optimal release profile for oxytocin brain delivery.

## 1. Introduction

Encapsulation of drugs in a nanoparticle has become a crucial component of drug delivery, translational pharmaceutical development, and research [1]. Drug delivery systems include micro and nanomaterials with numerous applications in therapeutics, diagnosis, imaging, gene delivery, and tissue engineering [2,3]. This application has been useful in delivering drugs with poor aqueous solubility and low bioavailability [4,5]. Moreover, compounds that are vulnerable to chemical and/or enzymatic degradation, such as peptides, are also great candidates for this delivery system [6]. For targeted delivery in managing brain disorders, drug molecules and peptides are required to penetrate the blood–brain barrier [7]. The ability of these molecules to travel across the impermeable barrier is one of the major concerns in translational research.

For example, although oxytocin neuropeptide is linked with various physiological functions in the central nervous system, e.g., social behavior and the peripheral organs, it has been reported as a potential therapeutic agent for seizure control and other brain disorders. However, its therapeutic potential is limited [8,9]. Oxytocin peptide consists of nine amino acid residues synthesized in the hypothalamus in mammals [10]. Oxytocin’s therapeutic potential in seizure control and other central nervous system disorders is often limited by its short half-life, rapid enzymatic degradation, and poor blood–brain barrier permeability [11]. Encapsulating oxytocin within polymeric nanoparticles can protect the peptide from premature degradation, enable sustained release, and enhance its bioavailability in the brain. Consequently, this approach holds promise for improving oxytocin delivery to the brain, potentially enhancing its therapeutic efficacy. On the other hand, the brain, being a critical organ, requires stringent homeostatic regulation and protection from both endogenous toxins and exogenous harmful agents present in the blood. To achieve this, the blood–brain barrier (BBB) has evolved as a highly selective and nearly impermeable interface. In addition to physical barriers such as tight junctions that limit paracellular permeability, the BBB employs active efflux transporter systems such as P-glycoprotein (Pgp) that function as a detoxification mechanism to restrict nonparacellular transport [12]. As a result, for a drug to successfully penetrate the brain parenchyma, it must be sufficiently small, moderately lipophilic, and not recognized as a substrate by these apical efflux transporters.

Interestingly, polymeric nanoparticles have extensively and successfully been used in the delivery of chemotherapeutic agents and anti-inflammatory and antioxidant drugs [13,14,15]. Bovine serum albumin (BSA) is an example of a biocompatible, non-toxic, non-immunogenic, and biodegradable carrier system for drug and antigen delivery [16]. Additionally, it has a sustained-release property and forms particles of small sizes, making them an attractive carrier systems [17,18]. One of these chemotherapeutic agents and carrier systems has been approved for use by the US Food and Drug Administration [19]. In the context of formulation, polymeric nanoparticles can be prepared by different methods ranging from nanoprecipitation, emulsification and solvent evaporation, and phase inversion, to solvent diffusion with sizes between 10–1000 nm [1,20]. However, these methods are devoid of precision and optimum control over the size distribution and uniformity, especially for large-scale processes [21,22]. In this regard, formulation methods and experimental conditions should be carefully selected.

In targeted delivery, rabies virus glycoprotein is a well-established brain-targeting ligand that binds to nicotinic acetylcholine receptors (nAChRs), which are highly expressed on neuronal cells and spread throughout the brain [23]. Conjugating RVG to bovine serum albumin (BSA) nanoparticles enables receptor-mediated transcytosis, facilitating efficient peptide transport across the BBB while reducing systemic off-target effects. By conjugating RVG to BSA nanoparticles via an amide bond formation, this system enhances brain-specific drug accumulation, offering a potential platform for treating central nervous system disorders.

In recent years, microfluidic synthesis of nanoparticles has provided a successful approach in a reproducible and reliable manner [24]. The microfluidics system is based on the laminar flow of liquids within micron-sized channels and the resulting hydrodynamic flow focusing of the solvent system [15]. Microfluidics has the possibility of being integrated with many other processes and technology [25]. Further, the system is reproducible, amenable to modifications, and economical [26,27]. The microfluidic device chip of different geometry is an essential and obvious characteristic of a microfluidic system which allows for fast analysis, small sample volume, and high surface area and provides a precise and controlled setting resulting in reproducible process parameters and outcomes. One of the most promising advantages of the microfluidics system is the integration of high-throughput particle synthesis. This capability makes it a powerful tool for developing micro- and nano-sized drug delivery systems (DDSs) with precise control tunable physicochemical properties, co-loading of multiple therapeutics, and functionalization with targeting moieties. With these unparalleled advantages, microfluidics has emerged as an effective approach to address questions in peptide delivery to the brain. The dynamics of cerebral fluids (i.e., blood and cerebrospinal fluid) play a crucial role in brain function, and significant efforts have been dedicated to advancing in vitro brain-on-a-chip models and microfluidic-integrated multifunctional devices. These innovative platforms are essential for studying neurophysiological processes, disease mechanisms, and therapeutic interventions. Conversely, conventional small-scale laboratory synthesis techniques suffer from batch variation and significant preparation times [28]. Han et al., demonstrated a microfluidics method for the encapsulation of a small peptide using PLGA as the polymeric matrix [6]. In another report using a microfluidic reactor, polymeric nanoparticles containing ribavirin were synthesized with sizes ranging from 50 to 200 nm [13]. For the formulation of nanoparticles, Gourdon et al., demonstrated both double and single emulsions for encapsulating oxytocin using PLA-PEG nanoparticles. However, this method is limited by poor drug loading and low initial burst release profile [29].

It has been previously demonstrated by our group that nanoparticle-based oxytocin formulated by the conventional nanoprecipitation method allowed for sustained delivery of oxytocin to the brain [30]. Furthermore, we have reported from our lab that nanoparticle encapsulation of oxytocin increased its blood–brain barrier permeability and duration of action [31,32]. The encapsulation of oxytocin enhanced its blood–brain permeability because the size of the nanoparticles was less than 200 nm. Encapsulating oxytocin in nanoparticles smaller than 200 nm significantly enhances its permeability across the blood–brain barrier (BBB). This size enables the particles to exploit transcytosis while also reducing clearance by the reticuloendothelial system and protecting oxytocin from enzymatic degradation. Moreover, the high surface area-to-volume ratio of these nanoparticles facilitates efficient transport into brain tissue, ultimately enhancing therapeutic efficacy for brain disorders. Using our nanoparticle-encapsulated oxytocin (NP-OT), we observed robust protection against induced seizures and restoration of social behavior in a *Scn1a*-derived mouse model [32]. Other researchers have explored the therapeutic potential of exogenous administration of oxytocin and neuropeptide in mitigating some CNS abnormalities [8,33,34,35]. Based on the previous efforts of our research group and other researchers, exploring the microfluidic-assisted formulation and optimization of BSA-loaded Oxytocin nanoparticles has not yet been reported. It is also not clear if a small particle size achieved from the microfluidics system would improve the release profile, and encapsulation efficiency, facilitate conjugation efficiency, and enhance biological activity. Herein, we hypothesize for the first time that the microfluidic-based approach could be used to obtain specific sizes of nanoparticle-based oxytocin in a controlled manner.

**Figure 1:**
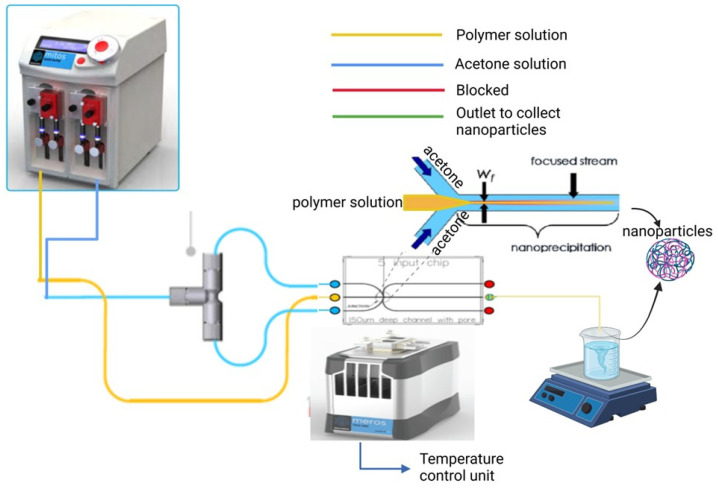
Schematic representation of the biphasic microfluidic system with the temperature control unit utilizing a 5-input flow focusing chip for nanoparticle synthesis using the nanoprecipitation method. Modified with permission from Dolomite. The figure was made with Biorender.com.

In this study, we investigated the effect of total flow rate, flow rate ratio, and polymer concentration on the particles’ size and polydispersity index. Additionally, the effect of the binary organic solvent mixture polarity index was investigated. Drug encapsulation efficiency, release, and kinetics profile were characterized. The cytocompatibility of the nanoparticles was also demonstrated. We conjugated the fabricated BSA nanoparticles to rabies virus glycoprotein (RVG). The overall goal of this manuscript was to optimize a formulation using the microfluidics system ideal for targeted brain delivery of oxytocin. Figure 1 shows a typical setup of a microfluidic system.

## 2. Materials and Methods

### 2.1. Materials

Bovine serum albumin (BSA) (Fraction V), acetone (analytical grade), ethanol (analytical grade), glutaraldehyde (25% in water), sodium bisulfite, and Sulfo-NHS (*N*-hydroxysulfosuccinimide) were procured from Fischer-Scientific (Pittsburgh, PA, USA). Rabies virus glycoprotein (RVG) and oxytocin were procured from GenScript (Piscataway, NJ, USA). The oxytocin ELISA kit was procured from Enzo Life Sciences (ADI-900-153A, NY, USA). Five-input microfluidics chips and quad pumps were purchased from Dolomite (Royston, UK).

### 2.2. Fabrication of BSA Nanoparticles Using a 5-Input Microfluidic Chip

Nanoparticles were formulated using a biphasic microfluidic system (Dolomite, Royston, UK) with a 5-input chip (150 μm channel). The system consists of an aqueous pump and an organic pump connected to flow rate sensors that allow modification in the flow rate ratio and flow rate of the two phases. The flow rate of both phases can be adjusted within the range of 10–1250 μL/min. A T-connector was used to separate the organic phase into two lines as shown in Figure 1. The system consists of an inbuilt pressure sensor which was used to monitor the pressure of the pumps during the process which are fixed. The aqueous and organic pumps were loaded with 0.2 μm filtered BSA solution and acetone, respectively. Each experiment varies only one factor at a time in a sequential manner.

### 2.3. Effect of Polymer Concentration

To investigate the effect of polymer concentration on the size of nanoparticles and polydispersity index, three different polymer concentrations of BSA in acetone (5 mg/mL, 10 mg/mL, and 15 mg/mL) were used to formulate nanoparticles using the microfluidics system described above. Residual acetone was evaporated at room temperature after 5 h of stirring, and the nanoparticles were frozen at −80 °C for at least 2–3 h before lyophilization.

### 2.4. Effect of Flow Rate Ratio

The polymer concentration at 10 mg/mL (1% *w*/*v* solution) was fixed and the flow rate ratio of the aqueous phase to the organic phase was varied using the Flow Control Center Software (version 5.4.18) from 1:1, 1:1.5, 1:3, 1:5, 1:7. Nanoparticles were collected and the polydispersity index and size were analyzed.

$$Flow\;rate\;ratio=\frac{Flow\;Rate\;(aqueous\;phase)}{Flow\;Rate\;(organic\;phase)}$$

### 2.5. Effect of Total Flow Rate

The polymer concentration and the flow rate ratio were fixed and the total flow rate for the formulation of the nanoparticles varied from 800 μL/min to 2000 μL/min. The size and the polydispersity index were analyzed.

### 2.6. Effect of Binary Organic Solvent Mixture

The polymer concentration, flow rate ratio, and total flow rate were fixed but the % of acetone in the organic binary solvent mixture polarity index was varied from 100% to 20%.

### 2.7. Formulation of OT-Loaded BSA NP Using a 5-Input Chip

After the optimization to this point, the encapsulation of oxytocin was conducted. Oxytocin (10% loading) was dissolved in 10 mg/mL of polymer (BSA) solution containing 10 mM NaCl; flow rate ratio (FRR) of 1:1.5 (aqueous phase to organic phase) and total flow rate(1500 μL/min) were used. The aqueous–organic phase solvent exchange happened within the output channels for the nanoprecipitation of nanoparticles to occur. Nanoparticles were subsequently collected into a beaker while stirring and the acetone was evaporated within 5 h. The nanoparticle suspension collected was crosslinked with 25% *w*/*v* glutaraldehyde in water overnight and the excess glutaraldehyde was neutralized with sodium bisulfite. The unloaded oxytocin was removed from the nanoparticle suspension by centrifugation at 15,000 rpm, leaving free oxytocin in the supernatant. The pellet was washed three times with distilled water to remove residual free oxytocin.

### 2.8. Conjugation of BSA Polymeric Nanoparticles to Rabies Virus Glycoprotein (RVG)

Firstly, the terminal groups of the carboxylic acid were activated using 1-ethyl-3-(3-dimethyl aminopropyl) carbodiimide (EDC). Here, 1 mg/mL of EDC and 1 mg/mL of NHS was added to the nanoparticle suspension. The mixture was constantly stirred for one hour. This way, the carboxylic acids were converted during the reaction to amine-reactive Sulfo-NHS esters. Secondly, the activated carboxylic acid terminal was treated with 1 mg/mL of rabies virus glycoprotein (RVG) for 2–3 h with constant mixing to form a stable conjugate amide bond formation. Finally, the samples were pre-freezed for 2–3 h before lyophilization using the Labconco freeze dryer (Labconco Corporation, Kansas City, MO, USA). We obtained the (FT-IR) spectral output from the IRAffinity-1S spectrometer (Shimadzu Corporation, Kyoto, Japan) to report the conjugation efficiency and analyzed the peaks by the transmittance as a function of wave number in the range of 4000–600 cm^−1^.

### 2.9. Characterization of RVG Conjugated Oxytocin BSA Nanoparticles

The nanoparticle size and the polydispersity index were determined by dynamic light scattering (DLS) in triplicate using a Zetasizer (Malvern Instruments Inc., Westborough, MA, USA). Here, 50 μL of samples was diluted with 1.5 mL distilled water. The measurements were done at room temperature. Both the lyophilized and immediately prepared suspensions were analyzed by DLS.

### 2.10. Encapsulation Efficiency of Oxytocin in BSA Nanoparticles

The amount of oxytocin encapsulated with the BSA nanoparticles was measured by the full degradation method with little modification [36]. Briefly, 5 mg of the OT nanoparticles were weighed and degraded by a forceful attrition process using pestle and mortar in 1 mL of PBS and kept undisturbed for 1 h. After 1 h, the solution was centrifuged at 15,000 rpm for 15 min and the oxytocin content in the supernatant was measured using an oxytocin ELISA assay kit.

$$Content\;of\;oxytocin=\frac{Actual\;loading}{Theoretical\;loading}$$

### 2.11. In-Vitro Drug Release

The release profile of oxytocin (OT) from nanoparticles was evaluated in phosphate-buffered saline (PBS, pH 7.4). Briefly, 5 mg of nanoparticles were suspended in 1 mL of PBS and incubated at 37 °C under continuous shaking (100 rpm). At designated time points, the suspension was centrifuged at 15,000 rpm for 15 min, and a portion of the supernatant was collected for analysis. The withdrawn volume was then replaced with fresh PBS. The concentration of released OT in the supernatant was quantified using an OT ELISA kit. All experiments were conducted in triplicate. To analyze the release profile, different kinetics models were used to report the release kinetics.

### 2.12. Cytotoxicity Assay

The safety of the oxytocin BSA nanoparticles was assessed using an in vitro MTT Assay [37]. 50,000 dendritic cells were seeded on a 48-well plate and were incubated overnight at 37 °C for the cells to adhere. Varying concentrations from 1 mg/mL to 0.5 μg/mL of nanoparticles suspended in complete DMEM media were introduced to the plates in triplicates and incubated for 48 h. DMSO (50%) and untreated cells were used as positive and negative control, respectively. A total of 25 μL of 0.5 mg/mL of MTT reagent was added into each well and incubated for 1–4 h at 37 °C. The plate was protected from light and was observed until a purple precipitate was formed. We carefully pipetted out the media without aspiration before adding 150 μL of DMSO to dissolve the formazan crystals and the plate was shaken for 15 min. The absorbance of the formazan was determined at 570 nm using a plate reader and was correlated to the percentage of viable cells.

### 2.13. Statistical Analysis

One-way ANOVA was used to determine the significance of the difference in treatment groups using GraphPad Prism 9.4.1 software (GraphPad Software, San Diego, CA, USA). Data were expressed as mean ± SEM, n = 3. The post hoc Dunnett’s test was used for multiple comparisons. ns: non-significant, ** *p* ≤ 0.01, *** *p* ≤ 0.001, and **** *p* ≤ 0.0001. The statistical significance criterion was *p* ≤ 0.05.

## 3. Results and Discussion

### 3.1. Effect of Bovine Serum Albumin Concentration

This manuscript aimed to generate nanoparticles using a microfluidic system to achieve small particle sizes with low polydispersity. Our findings demonstrate that a two-inlet flow-focusing microfluidic device enables the formulation of nanoparticles with tunable sizes and high stability. As shown in Figure 1, two streams of organic solvents merge with an aqueous polymer solution at the chip junction, generating a stable laminar flow. This controlled flow regime is essential for reproducible nanoparticle formation with minimal batch-to-batch variation. Once the laminar flow is established, switching the pumps ensures consistent and reproducible flow rates, thereby enhancing the reliability of the nanoparticle formulation process. The ability to fine-tune particle size under these conditions highlights the potential of this microfluidic approach for scalable and reproducible nanoparticle synthesis. The polydispersity index (PDI) is an indicator of the distribution of particle size within a sample. A lower PDI indicates a more uniform set of particle or molecular size distribution, which is desirable in applications like drug delivery, where consistency impacts performance. Nanoprecipitation is achieved primarily by the interfacial turbulence induced during the solvent displacement and the concentration of polymer plays a vital role in influencing the size and polydispersity index of nanoparticles [38].

**Figure 2:**
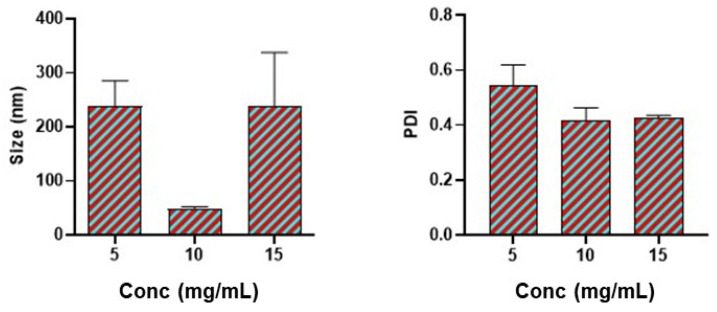
Impact of polymer concentration on particle size and polydispersity index (PDI). A polymer concentration of 1% yielded nanoparticles with an average size of 50 nm and a polydispersity index (PDI) below 0.4. Data are presented as mean ± SEM (n = 3).

The concentration of polymer affects the flow of fluids via the microfluidic chips. To this end, the concentration of bovine serum albumin was varied from 5 mg/mL to 15 mg/mL of the aqueous phase. Further, a concentration greater than 15 mg/mL clogged the chip due to agglomeration and there was no fluid flow. From Figure 2, the result shows that 5 mg/mL, 10 mg/mL, and 15 mg/mL polymer concentrations produced a size of nanoparticles approximately around 220 nm, 50 nm, and 220 nm respectively. Additionally, the polydispersity index (PDI) at 10 mg/mL of polymer was less than 0.40.

Approximately 50 nm nanoparticle size is desirable for brain permeability and the polydispersity index (PDI) was less than 0.4 which is optimal. In agreement with prior findings that nanoparticles around 50 nm are optimal for brain permeability, their in vitro data demonstrated that phenytoin sodium-loaded nanostructured lipid carriers (NLCs) with sizes below 50 nm exhibited an immediate drug release profile. This rapid release is essential for the prompt management of acute seizures [39]. Moreover, we found a nonlinear relationship between the concentration of BSA and the size of the nanoparticles. Similarly, the same relationship exists between concentration and the polydispersity index of nanoparticles. Several authors have observed that increasing the concentration of either BSA or PLGA polymer increases the size of the nanoparticles indicating a linear relationship [14,40,41]. However, Wang et al., reported an inverse relationship between the concentration of polymer from 10 mg to 50 mg/mL and the size of nanoparticles formulated by the coacervation method [42]. Again, Rahimnejad et al., observed a non-linear relationship between the concentration of polymer and the size of the nanoparticles [43] which was consistent with our result. The dynamics in this relationship is probably due to the interplay of different parameters affecting nanoparticle size for each designed study. Besides these, microfluidics chip geometry type, anti-solvent type, and volume could also play a major role in affecting the relationship between the concentration and the nanoparticle size as shown in Figure 2.

### 3.2. Effect of Flow Rate Ratio

**Figure 3:**
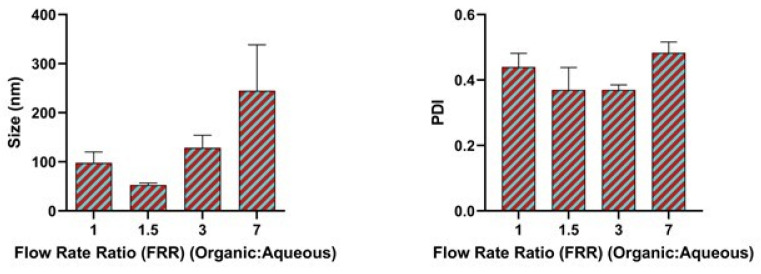
Impact of flow rate ratio on particle size and polydispersity index (PDI). The FRR of 1.5 produced particles of 50 nm in size with a polydispersity index (PDI) of less than 0.4. Data are presented as mean ± SEM (n = 3).

The flow rate ratio of the aqueous solution and the organic phase plays a vital role in influencing the laminal flow of fluids at the junction where the streams of solution converge which in turn affects the mixing process. We varied the flow rate ratio of the organic phase to the aqueous phase from 1 to 7. When the flow ratio was 1.5, a very small size of nanoparticles approximately less than 100 nm was observed as shown in Figure 3 which could be due to the efficient and rapid mixing process. Additionally, a polydispersity index (PDI) of less than 0.4 was observed. The higher flow rate ratio investigated produced a larger size nanoparticle and a high PDI > 0.4.

At a higher flow rate ratio, there was an increase in the mixing time between the two phases. Wang et al., also suggested that an increase in the size of nanoparticles at a high flow rate ratio could be due to the swelling of the nanoparticles resulting from the use of the larger volume of solvent [44]. It is well known that the non-solvent nanoprecipitation process involves supersaturation, nucleation, growth, and coagulation [45]. In addition, the degree of supersaturation achieved from the flow rate ratio can affect the final size of the nanoparticle obtained at a flow rate ratio of 1.5. In nanoprecipitation, precipitation is induced by the mixing of solvent and anti-solvent phases with resultant solvent displacement [46,47]. The mixing efficiency between the aqueous phase containing the polymer and the organic anti-solvent phase is crucial in regulating nucleation uniformity and rate, significantly influencing particle size, polydispersity index, encapsulation efficiency, and release profile [25,48,49].

### 3.3. Effect of Total Flow Rate

**Figure 4:**
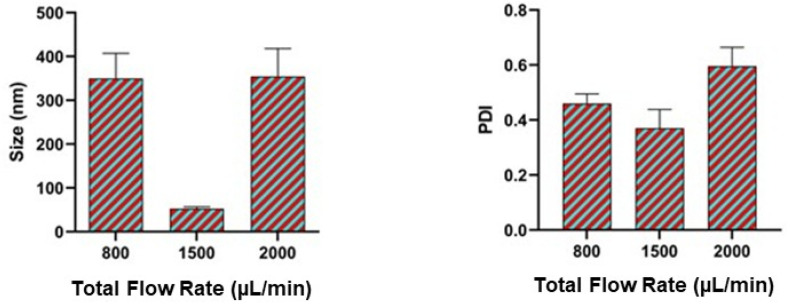
Impact of total flow rate on particle size and polydispersity index (PDI). The TFR of 1500 μL/min produced particles of 50 nm in size with a polydispersity index (PDI) of less than 0.4. Data are presented as mean ± SEM (n = 3).

The effect of total flow rate on the size of nanoparticles and polydispersity index are presented in Figure 4. The flow rate of the aqueous phase and the organic phase were controlled by using the automated system of the syringe pump. The efficiency of the mixing of the two phases in a hydrodynamic flow-focusing process affects the size of the nanoparticles. From our result, as shown in Figure 4, the total flow rate of 800 μL/min, 1500 μL/min, and 2000 μL/min produced a nanoparticle size of 350 nm, 50 nm, and 350 nm, respectively.

A total flow rate of 1500 μL/min produced a very small-sized nanoparticle. A total flow rate less or greater than 1500 μL/min produced a polydispersity index greater than 0.4. Moreover, when the total flow rate was increased from 1500 μL/min to 2000 μL/min, the Polydispersity index increased from <0.4 to approximately 0.6. Generally, it is believed that the higher is the flow rate, the higher is the shear stress and the smaller is the size of nanoparticles synthesized [50]. It is also believed that at higher flow rates, the volume of solvent used is reduced and the time of synthesis is also reduced. There is a possibility of chip integrity loss at a high flow rate, and this was also observed by Mares et al. [15]. Additionally, Hakala et al., demonstrated a microfluidic co-flow route for human serum album-drug nanoparticle synthesis [51]. In their findings, they identified that the higher the flow rate, the higher is the concentration of the organic solvent in the resulting mixture, and the bigger is the nanoparticles formed. This is in agreement with our results that the effect of each instrument parameter is specific to each experimental design.

### 3.4. Effect of Binary Organic Solvent Mixture

**Figure 5:**
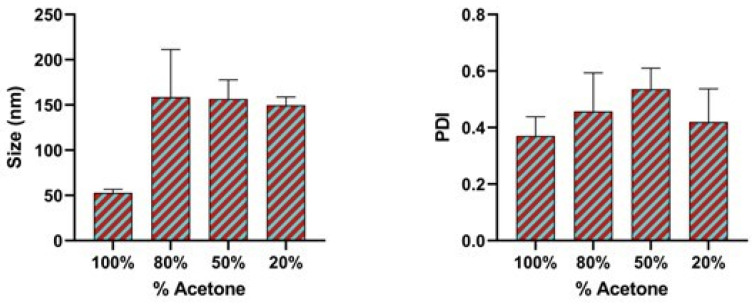
Effect of the binary organic solvent mixture (acetone and ethanol) expressed as the percentage of acetone on particle size and polydispersity index (PDI). Data are expressed as mean ± SEM (n = 3).

Organic solvents play a very critical role in the synthesis and assembly of nanoparticles even if all other factors are not changed. Additionally, liquid viscosity coupled with the interactions between the molecules of the solvent and the nanoparticle surface affects the final size of the nanoparticles. Therefore in our process of optimization, we attempted to modify the organic solvent and the organic binary solvent mixture polarity index by the addition of ethanol in varying volume ratios. As shown in Figure 5, altering the polarity index of the organic solvents increases the size and the polydispersity index of the nanoparticles. Acetone (100%) in the organic phase produced a size of 52.89 nm and a PDI of 0.37.

As the amount of acetone in the mixture was reduced there was a significant increase in the size of the nanoparticles and the polydispersity index (PDI). Acetone (80%)/ethanol (20%) and acetone (50%)/ethanol (50%) produced a size of 158.76 nm and 156.64 nm respectively. When a binary organic solvent was used, the interaction between the two solvents alters key parameters, such as effective polarity, viscosity, and miscibility with the aqueous phase, compared to a single solvent system. This modification affects the rate of solvent diffusion and the supersaturation level in the aqueous phase, leading to a slower and less abrupt nucleation process. Consequently, fewer nucleation sites form, allowing more time for individual particles to grow, which results in larger nanoparticles. Additionally, the altered interfacial tension in a binary solvent mixture may further promote coalescence, contributing to increased particle size.

The same approach was used in the synthesis of gold nanoparticles reported by Hussain et al. [52]. From their studies, 20% ethanol/80% water solution produced a nanoparticle size of 22 nm. Indeed, by controlling the solvent polarity index, different sizes of the nanoparticles can be achieved. Our results are in agreement with other studies where larger nanoparticles are synthesized by using either purely ethanol or ethanol mixture [53]. Mohammad-Beigi H. et al., also demonstrated the synthesis of human serum albumin nanoparticles with approximately a size of 100.11 nm by the desolvation method using only acetone in the organic phase [54]. However, in our case, the binary organic solvent mixture did not achieve the desired size in our optimization process though the nanoparticle sizes are less than 200 nm. This is possibly due to the hemiacetal formation between the alcohol and acetone which makes the acetone non-readily available for nanoprecipitation and also the differential miscibility of different organic solvents with water. Table 1 shows the different binary organic solvent mixture polarity index of the different ratio and their corresponding size of nanoparticles in each case.

Acetone (100%) was used for further formulation development because it can be fully removed at room temperature and more importantly, gave a small-sized nanoparticle. The mixing efficiency between the aqueous phase containing the polymer and the organic anti-solvent phase is crucial in regulating nucleation uniformity and rate, significantly influencing particle size, polydispersity index, encapsulation efficiency, and release profile [25,48,49]. Furthermore, Moran et al., demonstrated a new method of synthesizing peptide-loaded polymeric nanoparticles using non-aqueous nanoprecipitation [55]. These nanoparticles are prepared under strictly anhydrous conditions and many organic solvents are involved. However, one of the drawbacks of this method is ensuring the complete evaporation of the multiple solvents of different polarities. Additionally, CJ Luo et al., reported a novel electrospray nanoprecipitation for preparing polymeric nanoparticles but this method may not be ideal for encapsulating peptides due to the application of an electric field [56].

### 3.5. Characterization of RVG Conjugated Oxytocin BSA Nanoparticles

**Figure 6:**
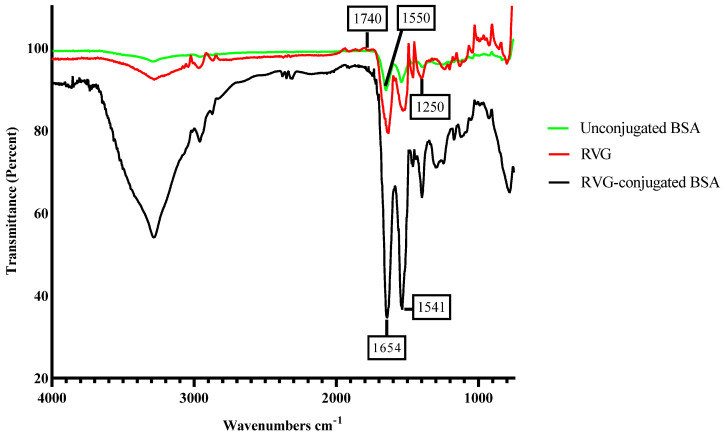
FTIR spectrum data collected to confirm the conjugation of the brain-targeting ligand (RVG) to the nanoparticles. Rabies virus glycoprotein (RVG) spectra show two main peaks that are linked with amide I and amide II vibrations at 1654 cm^−1^ and 1541 cm^−1^ respectively. These peaks were intense, and the amide vibrations indicated the conjugation of RVG (rabies virus glycoprotein) to bovine serum albumin (BSA).

Rabies virus glycoprotein (RVG) spectra show two main peaks that are linked with amide I and amide II vibrations at 1654 cm^−1^ and 1541 cm^−1^, respectively, in Figure 6.

These peaks were intense, and the amide intense vibrations indicated that the conjugation of RVG (rabies virus glycoprotein) to bovine serum albumin (BSA) was successful as shown in the spectra in Figure 6. For the unconjugated BSA nanoparticles, the amide I centered around 1740 cm^−1^ corresponds to the stretching mode of the CO bond of the amide. The amide peak II centered around 1550 cm^−1^ corresponds to the bending mode of the NH bond of the amide [30,57]. The bands of the characteristic functional group are present. One of the ways of delivering molecules to the brain is via the receptor-mediated process. Receptor-mediated transport is an alternative and innovative strategy for delivering compounds to the brain. Previously, we have used transferrin and rabies virus glycoprotein as brain-targeting ligands. Here, RVG, which is a short peptide that binds to nicotinic cholinergic receptors on the blood–brain barrier has been employed as a targeting ligand to deliver molecules to the brain. With this reliable active process, a wide range of biological molecules can be delivered to the brain shifting the paradigm of drug delivery to the central nervous system [58,59]. Given the relatively low brain-targeting efficiency of many ligands, off-target binding to endothelial cells in peripheral blood vessels can disrupt vascular permeability, triggering inflammatory responses due to endothelial activation. Additionally, nanoparticles and ligand-drug conjugates are often recognized by the reticuloendothelial system, resulting in undesired accumulation in the liver, spleen, and lymphatic system, which may lead to metabolic alterations. To mitigate these effects, enhancing ligand specificity is essential to minimize cross-reactivity with peripheral receptors and improve targeted drug delivery.

### 3.6. DLS Characterization of RVG Conjugated Oxytocin BSA Nanoparticles

After the optimization of the batch had been completed, the nanoparticles were analyzed by dynamic light scattering (DLS) in triplicate using a Zetasizer.

**Figure 7:**
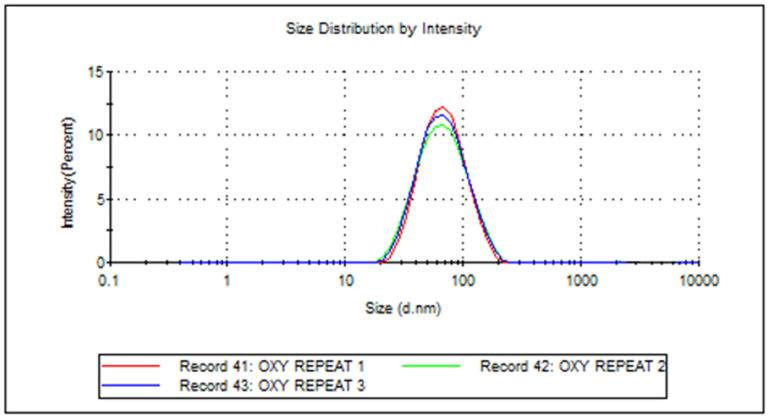
DLS characterization of RVG-conjugated oxytocin BSA nanoparticles formulated with a microfluidics system showing a uniform peak and size distribution.

The size of the nanoparticles is less than 100 nm with uniform peak and size distribution as shown in Figure 7. Nanoparticles less than 100 nm are optimal for brain permeability and drug release.

### 3.7. Encapsulation Efficiency of Oxytocin into BSA Nanoparticles

Lastly, after investigating how these parameters affected the nanoparticle’s size and polydispersity index engineered by the microfluidic system, the encapsulation of oxytocin into the polymeric matrix was addressed. Here, the goal was to explore if there is an improvement in the encapsulation efficiency of the nanoparticles fabricated by the microfluidic system with the 5-input chip and compare them with results from the conventional method in previous studies. The average encapsulation efficiency of oxytocin into BSA nanoparticles fabricated by the microfluidics system was 81.98 ± 0.3% *w*/*w*. These results show that the microfluidics-assisted preparation of BSA nanoparticles for oxytocin delivery is very efficient. The system is reliable, reproducible, and most likely due to the proper control of precipitation, laminar flow conditions, and short mixing time. In our previous study using the conventional nanoprecipitation method, the encapsulation efficiency of oxytocin into BSA nanoparticles was 75% ± 2.5% *w*/*w* [30]. Non-aqueous nanoprecipitation, single and double emulsion methods, and different polymeric matrix have been used to encapsulate oxytocin and other peptides with varying encapsulation efficiency [6,29,36,55]. To the best of our knowledge, this is the first time that a microfluidics system has been used to formulate BSA nanoparticles with higher oxytocin encapsulation. With the microfluidics system, the formulation is faster and material loss during the formulation process is minimal. On the other hand, The superiority of microfluidics over conventional techniques was demonstrated in a study by Vu et al. (2019) [60], which compared the fabrication of poly(lactic-*co*-glycolic acid) (PLGA) nanoparticles loaded with rutin using a bulk emulsion evaporation process and a micromixer-based microfluidic approach. The microfluidics-fabricated nanoparticles exhibited smaller particle sizes, a more uniform size distribution, higher drug entrapment efficiency, and faster drug release compared to those produced via bulk methods [60].

### 3.8. In-Vitro Oxytocin Release Profile

**Figure 8:**
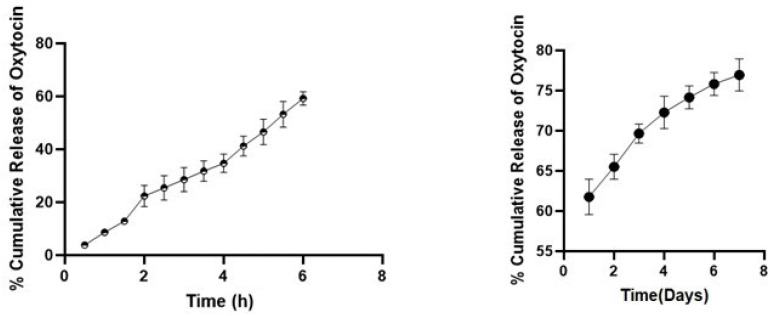
In vitro release profile of oxytocin from BSA nanoparticles fabricated by the microfluidics system in PBS at PH 7.4. Approximately 58% cumulative oxytocin release was achieved within 6 h and approximately 78% within 7 days. Data are expressed as mean ± SEM (n = 3).

To analyze the release profile of oxytocin from the BSA nanoparticles, the nanoparticles were suspended in phosphate buffer saline at PH 7.4, and aliquot samples were collected at a predetermined 30 min time intervals. To determine the initial burst release profile, release studies were conducted over a 6 h period with a 30 min sampling interval. As shown in Figure 8, after six hours, the percentage of cumulative oxytocin release was approximately 60%.

The sharp burst release of oxytocin from the BSA nanoparticles was due to the adsorption of the oxytocin on the nanoparticle surface. This has also been observed by others that higher burst release of molecules is achieved when molecules adsorb on the surface of nanoparticles due to large surface area [61]. We previously reported 35% cumulative release of oxytocin from BSA nanoparticles formulated by the conventional nanoprecipitation method [30]. Importantly, compared to the conventional nanoprecipitation method, we demonstrated an increased release profile of oxytocin from the BSA nanoparticles that were generated using the microfluidics system.

For the invitro release kinetics studies for the first six hours, different release models were evaluated. This includes % cumulative oxytocin release vs. time for zero order kinetics model; log % cumulative oxytocin release vs. log time for Korsmeyer–Peppas model; % cumulative oxytocin release vs. square root of time for Higuchi kinetics model; and log % cumulative oxytocin remaining vs. time for first order kinetics model [40]. From the best fit with the highest correlation value (R^2^) which was 0.9898 as shown in Table 2, we concluded that the nanoparticle-encapsulated oxytocin (NP-OT) followed the Korsmeyer–Peppas model.

Additionally, the magnitude of the release exponent n = 0.99 shows that the release mechanism is non-Fickian diffusion. This release mechanism involves a combination of diffusion through the matrix and polymer-controlled processes like swelling, erosion, or relaxation, leading to a complex release profile. Unlike Fickian diffusion, where drug transport is solely concentration gradient-driven, non-Fickian diffusion results in nonlinear release dynamics, often observed in polymeric systems such as nanoparticles.

### 3.9. Cytotoxicity Assay

**Figure 9:**
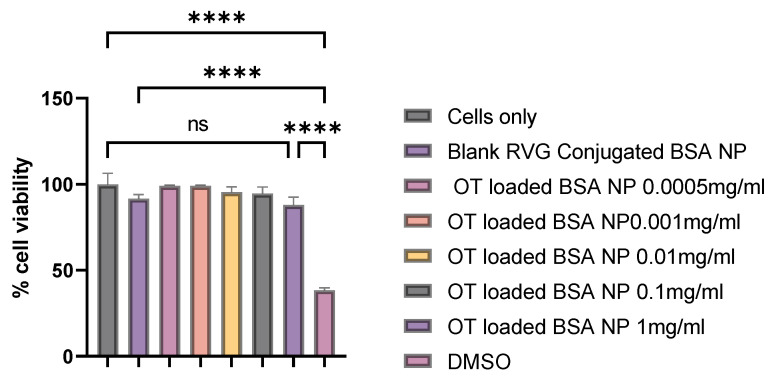
Cytotoxicity of nanoparticles engineered by the microfluidics system after 48 h of exposure to dendritic cells. The concentration of the nanoparticles was from 0.0005 mg to 1 mg/mL. Untreated cells and dimethyl sulfoxide (DMSO) were used as a negative and positive control, respectively. Data are expressed as mean ± SEM (n = 3). Statistical analysis was conducted by one-way ANOVA and post hoc Dunnett’s test was used for multiple comparisons. ns: non-significant, and **** *p* ≤ 0.0001.

To show that the BSA nanoparticles containing oxytocin engineered by the microfluidics system are safe resulting in therapeutic feasibility, we conducted a cytotoxicity assay. We observed no significant cell death after 48 h of exposure to different dilutions of the BSA nanoparticles which indicates that the preparation does not elicit any cytotoxic effects (Figure 9).

## 4. Conclusions

In the current manuscript, this study identifies 10 mg/mL BSA, FRR = 1.5, and a total flow rate of 1500 µL/min as the optimal parameters for generating the smallest particles using the microfluidics system. We generated BSA nanoparticles encapsulating oxytocin and found the nanoparticles to be non-toxic. Moreover, we demonstrated the small-size nanoparticles with improved encapsulation efficiency and the release profile of the oxytocin from the BSA nanoparticles compared to other previously published methods.

During our optimization process, A 5-input chip was used for the formulation. However, one of the challenges of microfluidics technology is that it is vulnerable to clogging. Therefore, further studies are warranted to improve the productivity of the microfluidic methods. In our follow-up study, we will compare the biological activity of our optimized microfluidics-assisted formulation with other methods of preparation in the in vivo preclinical studies. This report represents a crucial step in the formulation of therapeutics for brain disorders.

In conclusion, this study demonstrates the value of microfluidics technology in the synthesis of nanoparticles for oxytocin delivery to the brain.

## Figures and Tables

**Table 1:** The effect of binary organic solvent mixture polarity index on the size of the nanoparticles and the polydispersity index. The addition of acetone increases the size of nanoparticles and polydispersity index.

Binary Organic Solvent Mixture	Binary Organic Solvent Mixture Polarity Index	Size (nm)	Polydispersity Index (PDI)
100% Acetone solvent	5.2	52.89 ± 3.8	0.37
80% acetone + 20% ethanol	5.12	158.76 ± 52.5	0.4
50% acetone + 50% ethanol	5.15	156.64 ± 21.2	0.5
20% acetone + 80% ethanol	5.18	149.70 ± 9.05	0.4

**Table 2:** Analysis of the R-square values and rate constants of release kinetics of BSA nanoparticles containing oxytocin fabricated by the microfluidics system. The magnitude of the release exponent n = 0.99 shows that the release mechanism is non-Fickian diffusion.

Model	R^2^	K	n
Zero order	0.9884	8.67005	-
First order	0.8584	0.2647	-
Higuchi	0.9634	17.3401	-
Korsmeyer–Peppas	0.9898	0.0862	0.99

## Data Availability

The original contributions presented in this study are included in the article.
